# Exploring the Psychological Effects of Optimism on Life Satisfaction in Students: The Mediating Role of Goal Orientations

**DOI:** 10.3390/ijerph17217887

**Published:** 2020-10-28

**Authors:** Pablo Usán Supervía, Carlos Salavera Bordás, Víctor Murillo Lorente

**Affiliations:** 1 Departament of Psychology, Faculty of Human Sciences and Education, University of Zaragoza, Valentín Carderera, 4, 22003 Huesca, Spain; 2 Departament of Psychology, Faculty of Education, University of Zaragoza, Pedro Cerbuna, 12, 50009 Zaragoza, Spain; salavera@unizar.es; 3 Departament of Physiatry and Nursing, Faculty of Health and Sports Sciences, University of Zaragoza, Plaza Universidad, 3, 22002 Huesca, Spain; vmurillo@unizar.es

**Keywords:** optimism, goal orientations, life satisfaction, students, adolescents

## Abstract

Subjective wellbeing is a current issue today. Various variables affect subjective wellbeing during adolescence: a crucial stage in the life of the individual. This study focuses on analysing the relationship between academic goal orientation, optimism and life satisfaction in adolescent students, as well as the possible mediating role of the goal orientation (task and ego) in the relationship between optimism and life satisfaction in adolescents. **Methods:** The sample comprises 1602 students (male *N* = 871; 54.36% and female *N* = 731; 45.63%) from nine secondary schools. The instruments used were the Life Orientation Test Revised (LOT-R), the Perception of Success Questionnaire (POSQ) and the Satisfaction With Life Scale (SWLS) questionnaire. **Results:** The results of the study reveal significant correlations between optimism-related variables, goal orientation and life satisfaction. In addition, goal orientation was found to have a positive mediating role on optimism and life satisfaction. **Conclusion:** The study shows the importance of promoting adaptive behaviours in goal orientation in adolescents, leading to optimal levels on variables such as optimism and life satisfaction, which in turn improve the individual’s psychological development and academic performance.

## 1. Introduction

Research on quality of life has been approached from two broad perspectives: objective and subjective. While the objective approach focuses on the external circumstances of the subject, such as income levels, friendship networks and professional status, among others, the subjective approach centres on the individual’s point of view. Both perspectives will be explored in our study from three theoretical constructs in which we will investigate the relationship between them: optimism, life satisfaction and goal orientations.

In accordance with the above, the subjective judgements can refer to life in general or be concerned with individual-specific aspects [1]. One of these subjective judgements is optimism, which is defined as a more or less stable expectation to be heading towards positive or favourable circumstances [2]; conversely, pessimism is defined as the individual’s belief or presumption that unfavourable things are ahead [3]. Thus, optimism–pessimism is a one-dimensional construct that runs between two poles; that is, both concepts respond to a set of relatively stable features that can resist changes in environmental conditions, hence the label dispositional optimism [2].

According to Carver, Scheier and Segerstrom [4], optimism is a predisposition that mediates between the subject’s external setting and the way he or she interprets it. An optimistic viewpoint allows the individual to respond positively to adverse, critical and even traumatic circumstances, allowing he or she to overcome these difficulties with effort and determination. Likewise, whether a person will be optimistic or pessimistic depends on the person’s resilience and focus in assessing their own individual circumstances. Circumstances and their outcome, therefore, can be viewed from both an optimistic and pessimistic perspective based on perception.

The existing literature highlights the relationship between optimism and various psychological features. Optimistic individuals are more prone to adaptive behaviour [2], to have good expectations about future achievements [5] and to personal efficiency, [6] and less prone to intrapersonal vulnerability [7], personal unhappiness [8] and physical discomfort [9]. Vera-Villarroel, Pávez and Silva [10] suggest that there is a relationship between optimism and psychological and physical wellbeing, and argue that optimistic people are less prone to stress, exhaustion and cynicism than pessimistic individuals, and they also cope better with difficulties and become ill less often.

Optimism among adolescents has been paid relatively little attention. Ferrero and Rico [11] argue that better personal and social relationships in optimistic students can lead to better academic performance. Other studies link optimism with higher levels of self-concept, self-esteem, self-efficacy and assertiveness [12,13,14].

As such, optimism plays a key role in the person’s life, especially in infancy and adolescence, phases during which adult personality is established as a permanent dispositional feature [15]. While in infancy, optimism develops according to the people closest to the child as satisfactory or unsatisfactory situations occur, in adolescence, the cognitive capacities or abilities of students are revealed about their expectations and social comparison with their equals. Therefore, it is important to educate schoolchildren in optimism for adequate personal and moral development [15].

On the other hand, life satisfaction responds to cognitive components which, along with emotional components (positive and negative affects), constitute subjective wellbeing [16]. This construct reflects the way a broad array of vital circumstances are perceived. According to Diener [17], there is broad consensus regarding the basic characteristics of subjective wellbeing: (1) it is built upon each person’s life experiences and the way they are perceived; (2) it includes positive dimensions, not merely the absence of negative ones; and (3) it also includes broader perceptions about life in general.

Focusing on the cognitive component, life satisfaction is described as the general evaluation that individuals make about their own life and circumstances [18]. By making this evaluation, the subject examines tangible aspects of life, balancing good and negative things and reaching a conclusion about his or her level of satisfaction with life [16]. The affective component refers to positive and negative affects, relatively independent from each other. Affective balance refers to the emotions, moods and feelings that a person may experience. These can be all positive, all negative, or a combination of both positive and negative [16].

To date, research on life satisfaction has mostly focused on psychological and social risk assessment. This has proven to be valuable for predicting pathological states, the individual’s ability to cope with stressful situations and their effect on personal conduct [19]. Studies about life satisfaction in infancy and adolescence are few [20,21,22]. These studies suggest that optimism develops in different ways during these crucial stages [23,24].

Finally, concerning academic performance, the goal orientation [25,26] (Ames, 2002; Nicholls, 1989) approach currently stands as one of the most important social-cognitive theories, and is a widely used framework in educational psychology [25]. This theory addresses the reasons, goals and intentions that guide students in academic settings. In different achievement environments, such as school, the main target of students is to show their skills and capability. For this reason, those students oriented towards the task, in a more self-determined vision with more adaptive behaviours, tend to think that success in school comes from their own personal ability to carry out tasks by joining effort and motivation to their schoolwork while those students oriented towards the ego, in a less self-determined vision with less adaptive behaviours [27] think that success in school is the fact of feeling superior towards their classmates taking their capacity and personal competence in the development of their tasks in terms of clearly comparison and improvement, not coming from their personal ability to perform tasks.

Researches referring to goal orientations towards task have been related to the commitment of schoolchildren towards their school tasks in a particular way and the school in a general way [28]; intrinsic motivation in performing tasks [20]; students’ academic happiness [29] or even the development of coping strategies in extreme situations such as stress management in exams or anxiety in the face of new knowledge to be imparted [30]. Ultimately, task orientation is related to greater physical and emotional well-being of students [31]. In this way, those students with a clear display of ego are related to a lack of commitment and school dropout [32,33,34], feelings of anxiety in the performance of their tasks on a comparative level with their peers [35] as well as, in a generalized, with a low state of physical and emotional well-being [36].

The existing literature highlights the positive correlation between optimism and life satisfaction. Adolescents who are more satisfied with life are more optimistic and maintain positive relationships with their peers. Similarly, some studies suggest that students who are satisfied with life perform better in academic settings and present better mental aptitudes than pessimistic students [3], and are more prone to be on friendly terms with their peers [13]. Furthermore, life satisfaction can act as a deterrent to school dropout and can be used to predict the academic performance of adolescents [37].

Few studies link academic goal orientation and the above mentioned constructs. Some studies relate dispositional optimism and motivation. According to these studies, optimistic people are more prone to make an effort to achieve their ends, while pessimistic people are less committed and less willing to invest personal effort to achieve their goals [2,4,38].

In this context, and given the dearth of studies that deal with these variables jointly, this study aims to analyse the mediating role that academic goal orientation (task and ego) plays in the relationship between optimism and life satisfaction in adolescents, following Casas et al., [20] who highlight the scarcity of such studies in the existing literature. Two general hypotheses are put forward: (a) optimism is positively related to life satisfaction in adolescents, and (b) academic (task and ego) orientations will play a positive and negative mediating role, respectively, in the relationship between optimism and life satisfaction in adolescents.

## 2. Method

### 2.1. Sample

The study comprised 1602 students, both male (*N* = 871; 54.36%) and female (*N* = 731; 45.63%) (Table 1) from 9 public secondary schools. Their ages range from 12 to 17 years (M = 14.11; SD = 1.47). Participants were selected by simple random sampling. Inclusion criteria were the ability to read and communicate in perfect Spanish to make sure that they could understand and answer the questionnaire. Incomplete questionnaires were discarded and students with cognitive disorders who could not fully understand the questionnaire were excluded from the study.

### 2.2. Measurement Scales

Three widely used questionnaires were selected to channel the participant’s responses. First, level of optimism was measured with Scheier, Carver and Bridges’s Life Orientation Test Revised (LOT-R) [39], translated into Spanish by Ferrando, Chico and Tous [40]. The scale includes six items, three positive (e.g., “I am always optimistic about my future”) and three negative (e.g., “I never expect things to go my way”). Responses were given on a 5-point Likert scale ranging from “Strongly disagree” (1) to “Strongly agree” (5). The internal consistency (Cronbach’s α) of the translated questionnaire is 0.76, and 0.78 for our survey.

Goal orientations were measured with Roberts, Treasure and Balagué’s Perception of Success Questionnaire (POSQ) [41], translated into Spanish and validated by Martínez, Alonso and Moreno [42]. This questionnaire comprises 12 items that reflect the student’s goal orientation, six referring to task orientation (e.g., “In class I feel that I am successful when I work hard”), and six to ego orientation (e.g., “In class I feel that I am successful when I show my classmates and teachers that I am the best”). Responses were given on a 5-point Likert scale ranging from “Strongly disagree” (1) to “Strongly agree” (5). The reliability of this questionnaire in the school environment has been demonstrated in previous studies: Cronbach’s α was 0.85 for the task subscale and 0.82 for the ego subscale [41] and it was 0.85 and 0.84, respectively, in our study.

Finally, life satisfaction was measured with Diener, Emmons, Larsen and Griffin’s Satisfaction with Life Scale (SWLS) [43], translated and validated for Spanish adolescents by Pons, Atienza, Balaguer and García-Merita [44]. The scale comprises 5 items that measure degree of life satisfaction among participants (e.g., “I am satisfied with my life”). Responses were given on a 5-point Likert scale ranging from “Strongly disagree” (1) to “Strongly agree” (5). Internal consistency (Cronbach’s α) of the translated questionnaire is 0.84 and 0.86 in our study.

### 2.3. Procedure

The study was conducted with the cooperation of several secondary schools and the students’ parents/guardians’ informed consent. All participants and parents/guardians were informed about the nature of the study. Data collection took place on a single day in each educational centre. Thus, the directors or heads of studies of each institute were contacted to arrange the best day to carry out the questionnaires for the courses described. Subsequently, the tutors or teachers of the students assigned a part of a class to answer the questionnaires, which were always attended by one of the researchers. All the information was stored in a database to carry out the statistical analyses of the research. Therefore, all ethical guidelines issued in the Declaration of Helsinki [45] were met. The research protocol was endorsed by OPIICS research group (S46_17R), Psychology and Sociology Department, Universidad de Zaragoza. Questionnaires were treated anonymously, and participation was voluntary, with participants being allowed to abandon the survey half-way if they so wished. All subjects gave their informed consent for inclusion before they participated in the study.

### 2.4. Data Analysis

Descriptive statistics were used to characterise the social and demographic characteristics of the sample. Correlations between optimism and life satisfaction were calculated with IBM SPSS v26.0. software (IBM, Armonk, NY, USA). Finally, SPSS v26.0’s MACRO tool was used to carry out mediation analyses by bootstrapping (10,000 runs). For all the operations, a *p* ≤ 0 0.05 level of significance was adopted, with a 95% confidence level.

## 3. Results

### 3.1. Descriptive Variables

As illustrated in Table 2, males scored higher in optimism, ego orientation and life satisfaction; females yielded higher scores in task orientation.

### 3.2. Correlational Analysis between Optimism, Goal Orientation and Life Satisfaction

The correlations between the variables are presented in Table 3. All pairs of variables present significant positive correlations, but to different degrees. Optimism is positively correlated with goal orientation (task and ego); the strongest correlation is between optimism and life satisfaction (*r* = 0.523). Goal orientation (task and ego) are correlated with one another (*r* = 0.289) and satisfaction with life, and optimism is, somewhat more strongly, correlated with task orientation (*r* = 0.304).

### 3.3. Mediation Effects of Goal Orientation on the Relationship between Optimism and Satisfaction with Life

**Figure 1:**
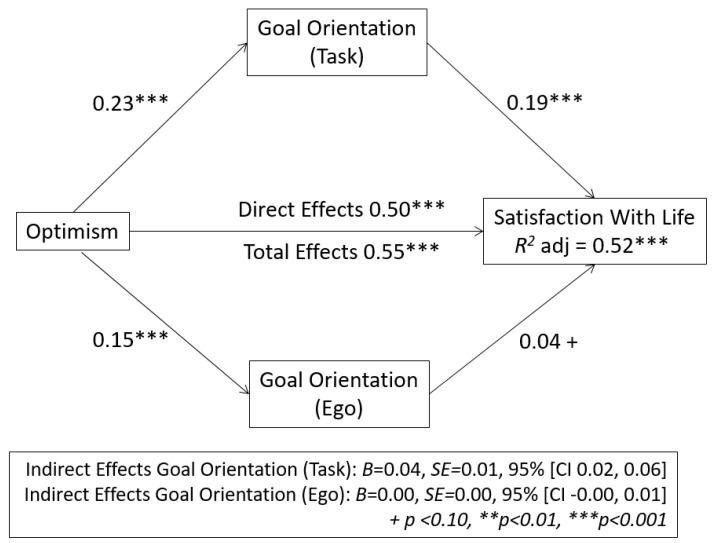
Mediation model of goal orientation in the relationship between optimism and satisfaction with life variables.

In order to assess whether the relationship between optimism and life satisfaction is mediated by goal orientation (task and ego), calculations were based on Tal-Or, Cohen, Tsarfati and Gunther [46] using Hayes [47] SPSS (v 26.0) Process 3.0 macro (Figure 1).

On the one hand, it was observed that task orientation mediated in the relationship between optimism and life satisfaction. The results indicate that optimism (VI) has an effect on the mediating variable (0.23), and this, in turn, has an effect on life satisfaction (VD) (0.19) (in both cases *p* > 0.001). Zero was not included in the bootstrap interval, *B* = 0.04, *SE* = 0.01, 95% (CI 0.02, 0.06), so it can be argued that task orientation mediates in the relationship between optimism and life satisfaction.

On the other hand, it was observed that ego orientation does not mediate in the relationship between optimism and life satisfaction. The results indicate that optimism (VI) has an effect on the mediating variable (0.15), but the effect of this on life satisfaction (VD) is not significant (0.04). Zero was included in the bootstrap interval, *B* = 0.00, *SE* = 0.00, 95% (CI 0.00, 0.01), so it can be argued that ego orientation does not mediate in the relationship between optimism and life satisfaction.

As such, in line with our hypothesis, optimism had a direct positive effect on life satisfaction (0.50, *p* < 0.001) and total effect (direct effect + indirect effect) (0.55, *p* < 0.001), which suggests that only task orientation mediates in the relationship between optimism and life satisfaction in adolescents, the proportion of variance being explained by model *R*^2^ = 0.52 ***.

## 4. Discussion

The aim of this study was to analyse the mediating role of task and ego orientations on the relationship between optimism and life satisfaction in adolescent students.

Our first hypothesis stated that optimism and life satisfaction are related in adolescents. Based on this, optimistic students ought to yield higher scores in the variables that reflect life satisfaction.

This hypothesis was fully confirmed; the correlational analysis shows that optimism and life satisfaction are moderately correlated, a result confirmed by the mediation analyses. This confirms that optimism increases the degree of life satisfaction in adolescents.

Previous studies point in the same direction. It has been argued that certain adaptive behaviours relate optimism and life satisfaction with other self-determined variables such as social adaptability, a positive outlook, greater resilience to adverse situations, subjective happiness, self-esteem and self-concept [3,48,49]. Similarly, various studies link optimism and life satisfaction with better school performance and lower rates of school dropout, again stressing these adaptive behaviours [50,51].

Our second hypothesis referred to the possible mediating role (positive and negative) played by task and ego orientations on the relationship between optimism and life satisfaction. This hypothesis was only partially confirmed. While task orientation plays a positive mediating role in the relationship between optimism and life satisfaction in adolescents, ego orientation was not found to play an equivalent role in the opposite direction, as we had hypothesised it would.

In any event, although ego orientation was found to have no impact on the relationship, the mediating role played by task orientation in the relationship between optimism and life satisfaction needs to be stressed.

Few studies have examined the mediating role of goal orientation on the constructs taken into consideration in this study. Some studies, however, have pointed out the positive effect of task orientation on various self-determined variables, such as persistence, will, and commitment to school tasks [20]; greater academic engagement and lower incidence of school burnout [52]; greater intrinsic motivation and better performance in carrying out of school tasks [53]; effort, performance, and greater academic joy and happiness [29]; and, more broadly, greater physical, psychological and emotional wellbeing [31].

Similarly, other studies relate motivational orientation with dispositional optimism, suggesting that more optimistic individuals are likely to invest more effort to achieve their ends than pessimistic individuals, who are less engaged and less likely to commit to their assigned tasks [2,4].

## 5. Conclusions

The results clearly indicate the positive relationship between task-oriented goals, optimism and life satisfaction in adolescents. At the same time, the task-oriented goal acted as a mediating variable between optimism and satisfaction with life, which accentuates the influence of this variable between both constructs. Although we observed the non-mediating effect of the ego-oriented goal between optimism and life satisfaction, we can affirm the bilateral correlational relation with both variables but not its mediating influence. Therefore, along with other personal and contextual variables, these constructs play a critical role in the configuration of the student’s personality and they directly affect their school performance and engagement with their educational centres. It is thus critical to promote these attitudes in both academic and family environments, facilitating students’ optimal personal and academic development. Finally, it is worth stressing that our results are but one step which encourages us to continue our research on the psychological characterisation of adolescence with the ultimate aim of contributing to the optimal personal, social and emotional development of adolescent students.

## Figures and Tables

**Table 1:** Results by students’ gender, age and academic year.

Sociodemographics		*N*	%
Gender	Male	871	54.36
	Female	731	45.63
Age	12 years	296	18.47
	13 years	284	17.72
	14 years	373	23.28
	15 years	387	24.15
	16 years	221	13.79
	17 years	41	2.55
Academic year	ESO Year 1	368	22.97
	ESO Year 2	436	27.21
	ESO Year 3	527	32.89
	ESO Year 4	271	16.91

**Table 2:** Results by descriptive variables optimism, goal orientation and life satisfaction.

Variables	Total		Male		Female	
	x	sd	x	sd	x	sd
Optimism	3.62	0.80	3.72	0.75	3.51	0.84
Goal orientation (Task)	3.80	0.81	3.71	0.82	3.91	0.76
Goal orientation (Ego)	2.89	1.03	2.95	0.98	2.82	1.08
Life satisfaction	3.52	0.85	3.60	0.82	3.43	0.89

**Table 3:** Results by correlational analysis of optimism, goal orientation and satisfaction with life variables.

Variables	Goal Orientation (Task)	Goal Orientation (Ego)	Satisfaction with Life
Optimism	1		
Goal orientation (Task)	0.232 **	1	
Goal orientation (Ego)	0.123 **	0.289 **	1
Satisfaction with life	0.523 **	0.304 **	0.161 **

** *p* < 0.01 (bilateral).
